# Case Report: Life-threatening pulmonary haemorrhage following hybrid VSD closure and pulmonary artery de-banding: major aortopulmonary collaterals as a hidden danger

**DOI:** 10.3389/fcvm.2026.1783754

**Published:** 2026-03-26

**Authors:** Stasa Krasic, Ivan Dizdarevic, Vesna Topic, Milan Rodic, Nemanja Djordjevic, Vladislav Vukomanovic

**Affiliations:** 1 Cardiology Department, Mother and Child Health Institute of Serbia, Belgrade, Serbia; 2 Faculty of Medicine, University of Belgrade, Belgrade, Serbia; 3 Cardiac Surgery Department, Mother and Child Health Institute of Serbia, Belgrade, Serbia; 4 Radiology Department, Mother and Child Health Institute of Serbia, Belgrade, Serbia; 5 Pulmology Department, Mother and Child Health Institute of Serbia, Belgrade, Serbia

**Keywords:** ECMO - extracorporeal membrane oxygenation, hybrid VSD closure, MAPCA-major aortopulmonary collateral artery, pulmonary artery debanding, pulmonary haemorrage

## Abstract

**Objective:**

Pulmonary haemorrhage (PH) after hybrid ventricular septal defect (VSD) closure has not been documented in the literature and is rarely reported after pulmonary artery debanding. We presented a 5-month-old infant with severe PH requiring extracorporeal membrane oxygenation (ECMO) support due to major aortopulmonary collater (MAPCA) after hybrid VSD closure and pulmonary artery de-banding.

**Case report:**

In a full-term female newborn [body weight (BW) 3.1 kg], prenatal diagnosis identified a muscular VSD and aortic coarctation. She underwent the first stage surgical repair through left lateral thoracotomy on day 6 of life. At six months (BW 5.1 kg), a hybrid VSD closure and pulmonary artery debanding were performed via median sternotomy, due to fatigue and poor weight gain. Post-procedurally, she developed massive PH, partially controlled with endotracheal adrenalin, tranexamic acid and NovoSeven®. As ventilation parameters required escalation, central venoarterial ECMO was initiated. During the 7-day period, circulatory support was maintained without complications, and, with improved ventilation, ECMO was discontinued. Four days later, the patient experienced significant tracheobronchial bleeding again, which was controlled with endobronchial adrenaline. CT angiography identified a MAPCA, and on the same day, cardiac catheterisation with embolisation was performed using Amplatzer Vascular Plug 4. The bleeding did not recur. As her condition on mechanical ventilation improved gradually, she tolerated the weaning process well.

**Conclusion:**

Clinicians should be highly vigilant for MAPCAs in patients experiencing massive pulmonary haemorrhage following pulmonary artery debanding. This case underscores the importance of preoperative screening for MAPCAs in infants undergoing staged repairs for coarctation and VSD, particularly when pulmonary artery banding appears successful, and oxygen saturation appears normal.

## Objectives

A two-stage repair for coarctation (CoA) with an associated ventricular septal defect (VSD) can achieve excellent early and mid-term outcomes. The initial stage comprises repairing the coarctation and performing pulmonary artery banding (PAB) to prevent congestive heart failure caused by the VSD. The second stage involves removing the pulmonary artery banding and closing the VSD (1). The muscular VSD (mVSD) are often hidden within coarse right ventricular trabeculations, making direct surgical visualisation via a typical right atrial approach difficult, potentially resulting in significant postoperative residual defects, or using a right ventricle approach that leads to ventricular dysfunction, tricuspid valve insufficiency, arrhythmias, and ventricular aneurysm formation (2). In suitable cases, minimally invasive percutaneous treatment can be performed, except for a standard surgical on-pump VSD closure. A typical minimum weight for standard percutaneous closure is 5 kg. Nonetheless, procedures have been successfully carried out within this weight range, with studies indicating success rates of approximately 80%–85% in this low-weight group, sometimes resulting in fewer complications compared to repeat surgeries (3, 4).

Hybrid VSD closure methods integrate minimally invasive surgical techniques, often via partial sternotomy, with interventional catheter techniques performed on a beating heart, thereby avoiding cardiopulmonary bypass. After partial sternotomy and exposure of the right ventricle, the puncture site was identified under TEE guidance. A purse-string suture was placed around the site, and the site was punctured with a trocar. The short sheath was then advanced through the defect into the left ventricle using the Selinger method. Finally, the occluder was deployed through the loaded sheath under TEE visualisation. This approach offers a less invasive alternative, with smaller incisions, reduced trauma by eliminating the need for a heart-lung machine, shorter hospital stays, and better cosmetic results compared with traditional open-heart surgery. The hybrid method not only reduces the risk of major complications compared with conventional surgery but also yields outcomes non-inferior to transcatheter occlusion in carefully selected patients with VSD (2, 5–7).

However, it also carries risks, including conduction blocks, residual shunts, left ventricular perforation, and device-related complications such as valve damage, air embolism, and haemolysis. These complications are particularly concerning in patients with complex congenital heart defects (2, 7). Pulmonary haemorrhage (PH) after hybrid VSD closure has not been documented in the literature and is rarely reported after pulmonary artery debanding (8, 9).

We presented a 5-month-old infant with severe PH requiring extracorporeal membrane oxygenation (ECMO) support due to major aortopulmonary collaterals (MAPCA) after hybrid VSD closure and pulmonary artery de-banding.

## Case report

**Figure 1:**
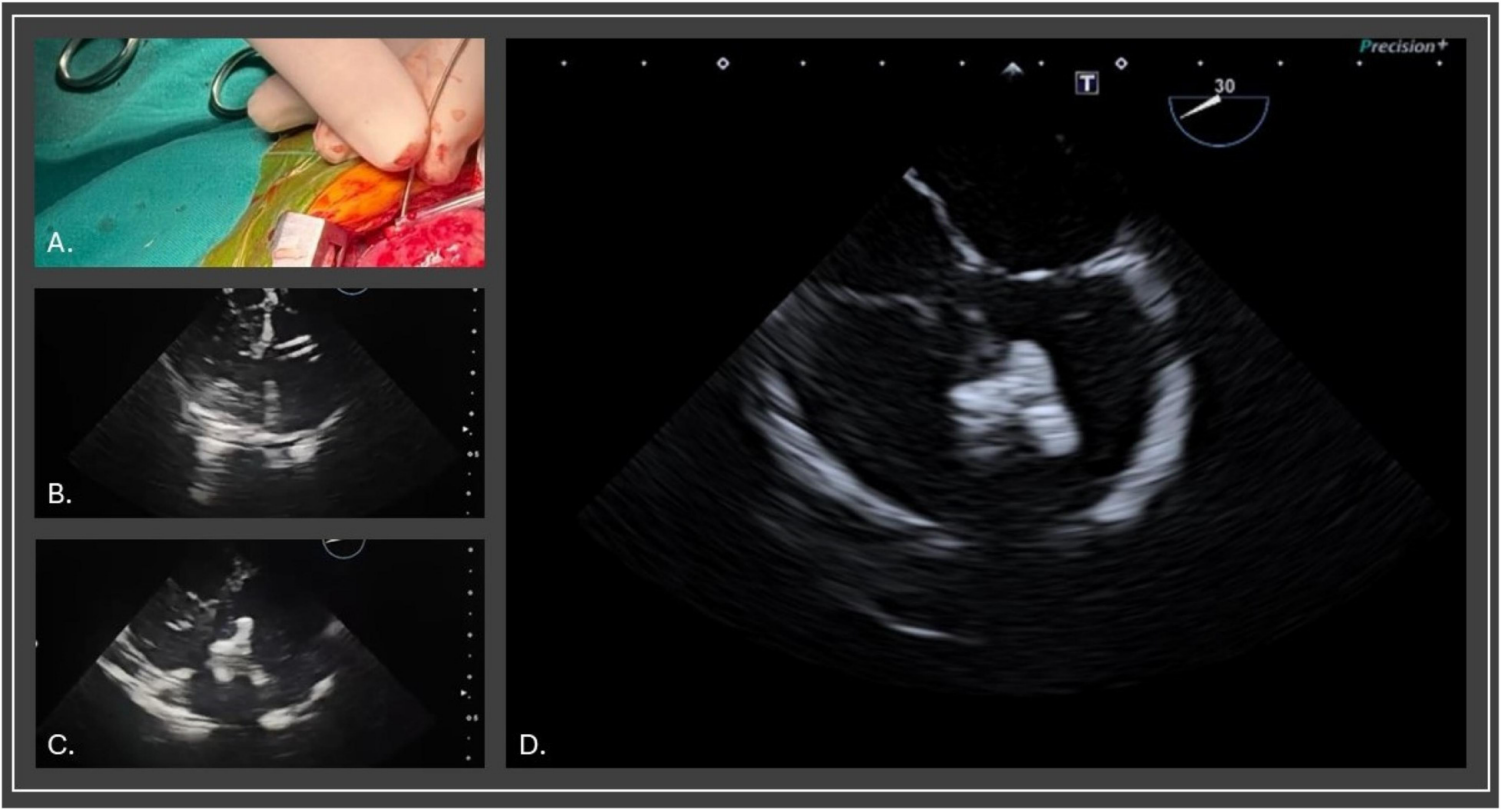
A right ventricular free wall puncture **(A)** allowed placement of a 6-F short sheath across the VSD into the left ventricle **(B)** transesophageal echocardiography revealed an amplatzer ductal occluder (ADO) 1 connected to the delivery cable **(C)** position of the ADO1 after deployment **(D)**.

**Figure 2:**
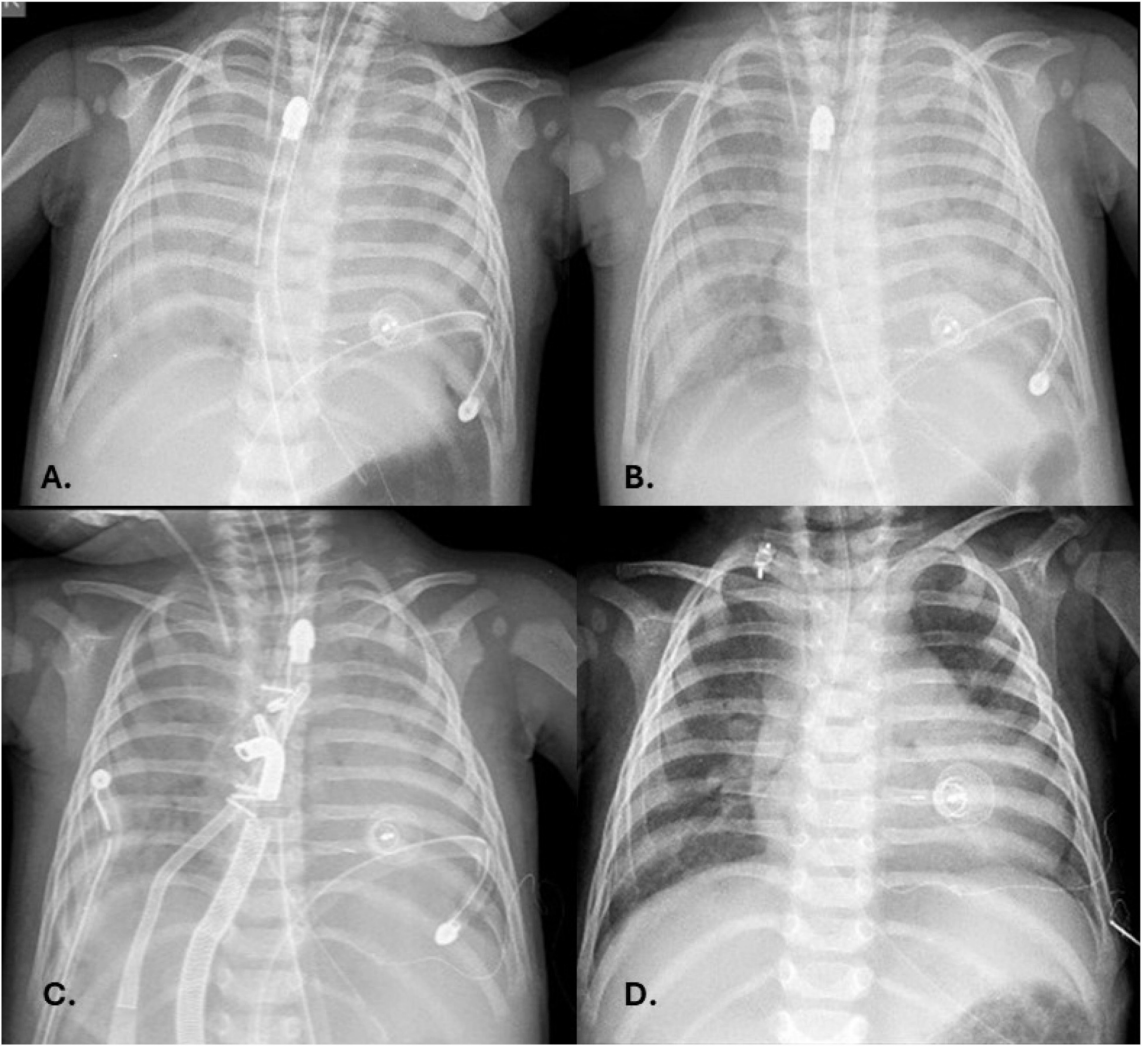
X-ray findings immediately after massive pulmonary haemorrhage **(A)**; day after **(B)**; after ECMO initiation **(C)**; on discharge **(D)**.

**Figure 3:**
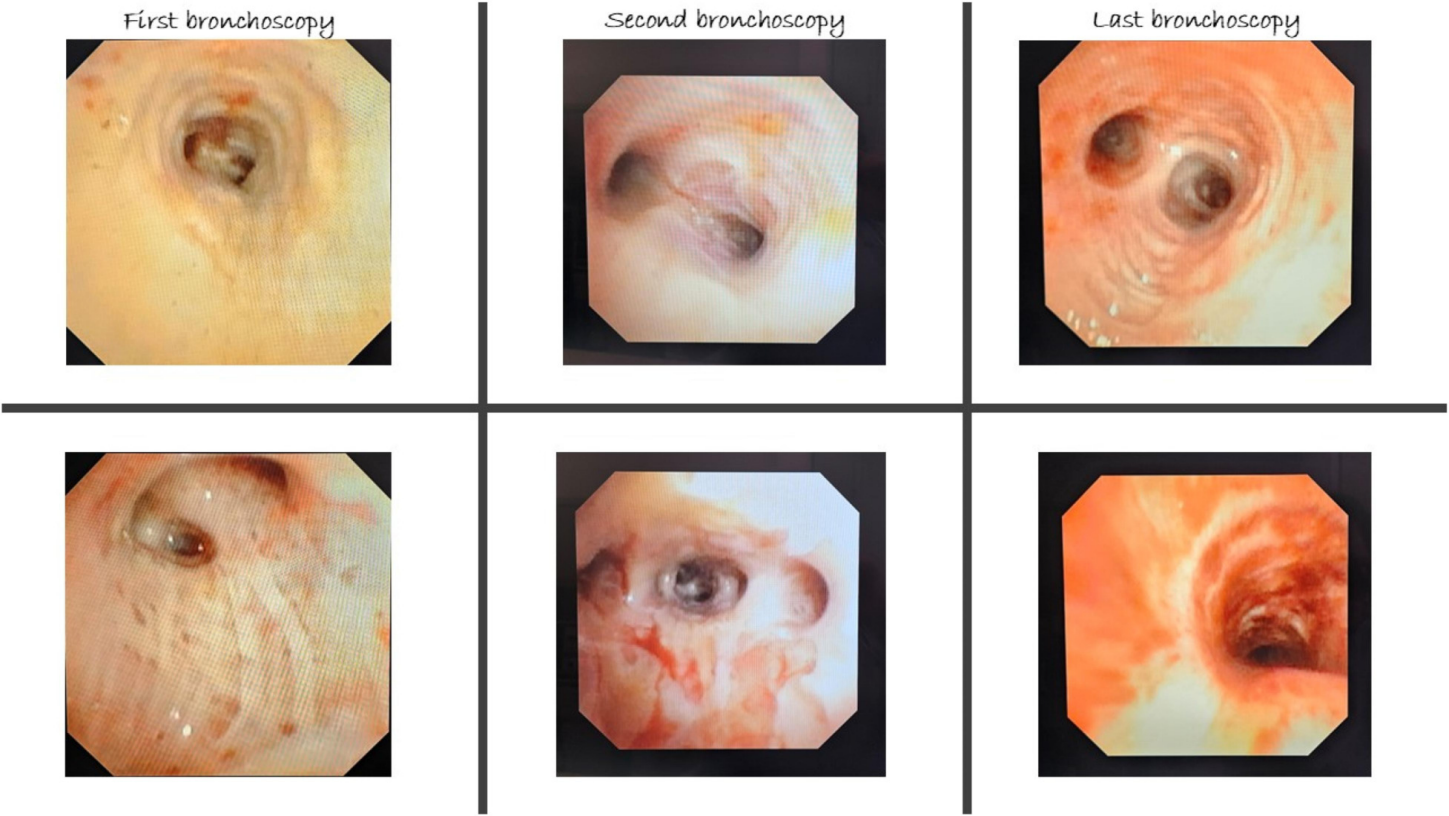
Bronchoscopy finding. First endoscopy: circumferential pale-yellowish pseudomembranes and fibrin stripes were seen in both trachea and main bronchi. Second endoscopy: scarce spots of friable mucosa within larger areas of pseudomembranes starting to appear in lobar and segmental bronchi. Last endoscopy: restored pattern of pale pink tracheal mucosa with larger areas of erosions.

In a full-term female newborn [body weight (BW) 3.1 kg], prenatal diagnosis identified a mVSD and CoA. She underwent the first stage surgical repair through left lateral thoracotomy on day 6 of life. Postoperative complications included bronchiolitis and poor growth. She was discharged on day 31 with a BW of 2880 g, and echocardiography showed an 8.5 mm muscular VSD, effective PAB with a 95 mmHg pressure gradient (PG), and corrected CoA. During the 5-month follow-up, she remained fatigued, with poor weight gain, and her oxygen saturation was approximately 96%. At six months (BW 5.1 kg), a hybrid VSD closure and pulmonary artery debanding were performed via median sternotomy. A right ventricular free wall puncture allowed placement of a 6-F short sheath across the VSD into the left ventricle. A purse-string suture was placed around the chosen location. Transesophageal echocardiography (TEE) guided the successful deployment of a 10 × 8 Amplatzer ductal occluder (ADO) 1 (Figure 1). The TEE detected no residual shunt and valve dysfunction. The banding tape was dissected and removed. Postoperative hydrocortisone was administered. Post-procedurally, she remained haemodynamically stable, intubated, with good arterial gas parameters. Eighteen hours later, she developed massive PH, partially controlled with endotracheal adrenalin, tranexamic acid and NovoSeven®. Due to persistent low oxygen saturation and ventilation difficulties despite high MV settings, high-frequency oscillatory ventilation (HFOV) was initiated. Chest x-ray showed bilateral lung opacities (Figures 2A,B). The following day, surfactant therapy was started. As ventilation parameters required escalation, central venoarterial (CVA) ECMO was initiated, with cannulas placed in the ascending aorta and right atrium. ECMO support was established smoothly, along with protective ventilation and a continuous heparin infusion. Regular echocardiography indicated good cardiac function and adequate left atrial volume. Neurological assessment was started after discontinuation of relaxation therapy on the second ECMO day. During ECMO, bronchoscopy removed large volumes of secretions containing fibrin deposits (Figure 3). Intratracheal alteplase, Pulmozyme, and surfactant were administered. Lung compliance improved gradually, reducing ventilation pressures, and subsequent chest x-rays showed progressive pulmonary recovery (Figures 2C,D). Over the following week, circulatory support was maintained without issues.

On the eighth postoperative day, fresh blood appeared in the drains, and despite stopping heparin, adequate anticoagulation was not achieved. The cannulation sites were revised twice, revealing no bleeding at the surgical sites. A bronchoscopy was conducted, removing a large amount of mucus plugs and old coagulum. With sufficient ventilation, the decision was made to discontinue ECMO support in the ICU. A gradual transition to full conventional mechanical ventilation was carried out, and the ECMO flow was decreased and turned off after 45 min. The patient maintained adequate gas exchange and ventilation, and systemic protamine was administered. Subsequently, decannulation was performed, and the chest was closed without hemodynamic issues.

**Figure 4:**
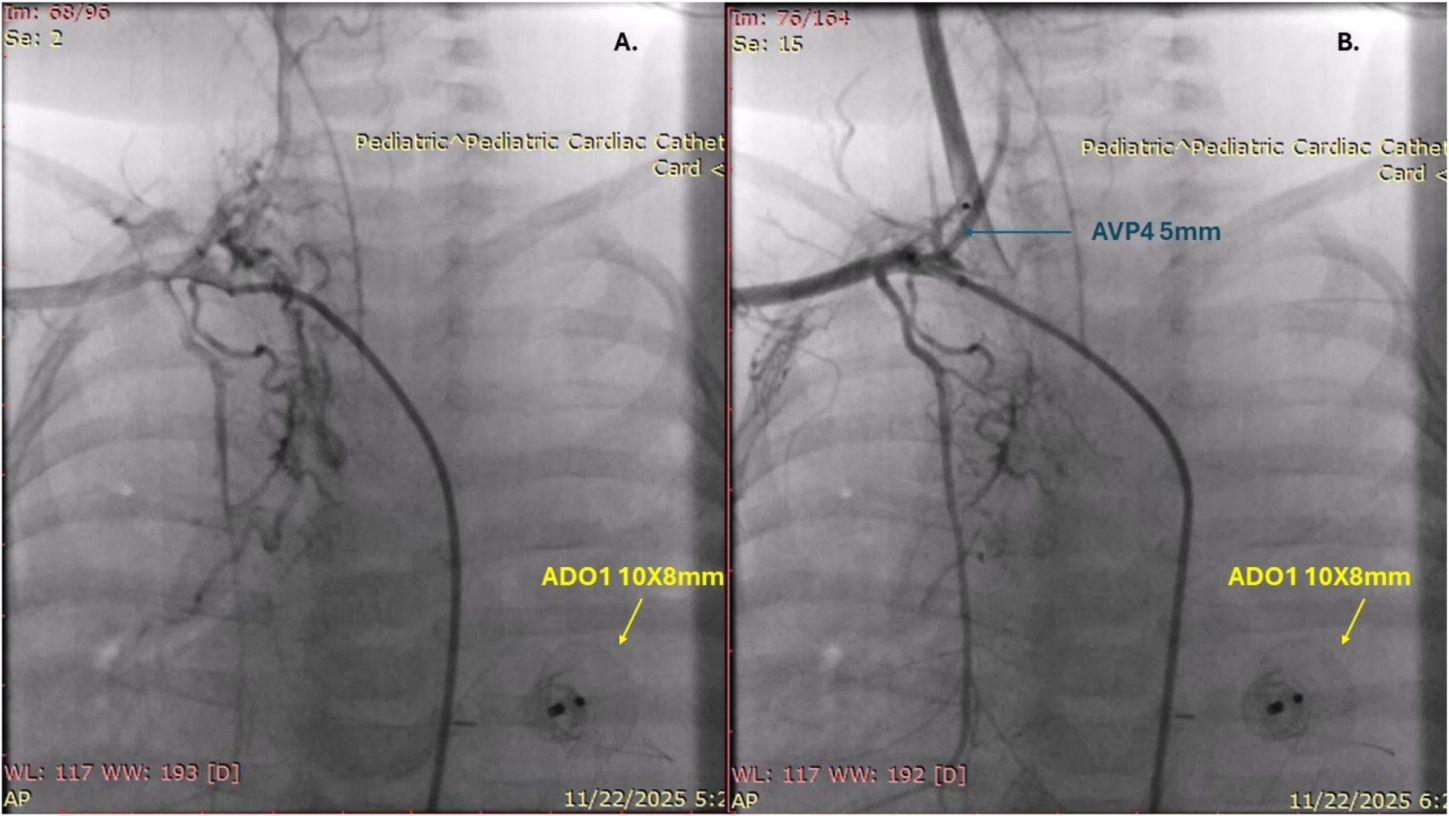
Massive major aortopulmonary colateral **(A)** occlusion using amplatzer vascular plug 4 **(B)**.

On the fifteenth day after surgery, the patient experienced significant tracheobronchial bleeding again, which was controlled with endobronchial adrenaline. She was relaxed and transferred to controlled mechanical ventilation. CT angiography identified a MAPCA, and on the same day, cardiac catheterisation with embolisation was performed using Amplatzer Vascular Plug 4 (Figure 4). Following the procedure, relaxation was discontinued, and she was switched to SIMV mode while maintaining adequate gas exchange. The bleeding did not recur. A subsequent bronchoscopy showed improved local conditions, with fewer fibrin deposits and less airway secretions. As her condition on mechanical ventilation improved gradually, she tolerated the weaning process well. She was extubated on the 25th postoperative day and started on non-invasive CPAP support. Over time, she transitioned from nasal cannula oxygen to complete weaning on the 32nd postoperative day and was discharged home on the 43rd postoperative day, weighing 5120 g.

## Discussion

Percutaneous VSD closure is possible in 5 kg infants, though it remains challenging. Advanced methods like periventricular (hybrid) techniques or using miniaturised devices are often chosen, offering a less invasive alternative to surgery- especially for certain defect types such as muscular or postoperative residual VSDs. The first off-pump periventricular device closure of a VSD was conducted in animal studies in 1997 under TEE guidance and was later performed on an infant with mVSD. Today, it is a common approach for VSD, especially in China. Literature reports that the perventricular approach successfully treats 88.5%–96% of mVSDs, including those in infants (2, 7). Considering our patient's weight, the size of the mVSD, and the desire to minimise trauma, a hybrid VSD closure with simultaneous pulmonary artery de-banding was chosen. Debanding is typically performed in conjunction with the surgical repair of the initial congenital heart defect, when patients reach an appropriate size or age (8). Our patient underwent de-bending in the 6th month of life due to fatigue and poor weight gain. Post-procedure, the patient developed severe PH, requiring intensive support with ECMO, despite previous full medical treatment (endotracheal adrenalin, tranexamic acid, NovoSeven®, surfactant), and HVOF.

Pulmonary haemorrhage, a rare complication after pulmonary artery debanding, can be linked to ischemia-reperfusion (IR) lung injury. In pig models, IR-related lung damage was significantly less if it occurred 5 weeks after pulmonary artery ligation, likely because the lung's systemic blood supply had time to develop, reducing ischemia (9). PH is a rare (0.01%–0.47%) complication following pulmonary artery stenting, with the mortality rate ranging between 50%–75%. The primary causes are vascular injury from stiff stents or IR injury when blood flow is suddenly restored to previously obstructed vessels. A great, rapid change in the systolic pressure gradient (SPG) > 34 mmHg during the procedure is a major predictor of injury (10). An acute lung reperfusion injury was observed in 22% of patients after balloon angioplasty for pulmonary artery stenosis (11). Additionally, the International Society of Heart and Lung Transplant (ISHLT) grading suggested that a PaO2/FIO2 ratio of 200 to 300 indicates ARI and a ratio of less than 200 indicates severe RI (11). Strategies to prevent IR lung injury include reducing ischaemia duration, using leukocyte-depleted blood reperfusion, employing heparin-coated circuits, administering prostaglandin E2, corticosteroid therapy, and N-acetylcysteine (12). Our patient underwent hybrid VSD closure and received corticosteroids post-procedure. Conversely, managing reperfusion injury focuses on lung-protective ventilation, inhaled nitric oxide, conservative fluid management, prone positioning, and ECMO (12).

Some studies indicate that MAPCAs can cause hemoptysis and PH after cardiac surgery in children with CHD (13, 14). Successful embolisation of MAPCA was key to controlling bleeding and aiding recovery in our patient. Therefore, MAPCAs might rarely cause significant PH following pulmonary artery de-banding in children. Removing the PA band suddenly increases blood flow and pressure in the pulmonary arteries. If large, existing MAPCAs transmit this surge and high systemic pressures directly to the lung's microvasculature. This sudden high pressure can rupture small vessels in the lungs, leading to severe PH. Additionally, even if an effective PAB were present, our patient had normal percutaneous oxygen saturation, which could suggest the presence of MAPCAs preoperatively.

To reduce the risk of postoperative complications and potentially prevent postoperative PH, preprocedural diagnostic methods such as CT angiography or cardiac catheterisation should be used, as they provide additional insight into the presence of MAPCAs.

Clinicians should be highly vigilant for MAPCAs in patients experiencing massive pulmonary haemorrhage following pulmonary artery debanding. This case underscores the importance of preoperative screening for MAPCAs in infants having staged repairs for coarctation and VSD, especially when PAB appears successful and oxygen saturation levels seem normal.

## Data Availability

The raw data supporting the conclusions of this article will be made available by the authors, without undue reservation.
